# First person – Susan M. Motch Perrine and Meng Wu

**DOI:** 10.1242/dmm.040568

**Published:** 2019-05-30

## Abstract

First Person is a series of interviews with the first authors of a selection of papers published in Disease Models & Mechanisms (DMM), helping early-career researchers promote themselves alongside their papers. Susan M. Motch Perrine and Meng Wu are co-first authors on ‘Mandibular dysmorphology due to abnormal embryonic osteogenesis in FGFR2-related craniosynostosis mice’, published in DMM. Susan is an Assistant Research Professor of Anthropology in the lab of Joan T. Richtsmeier at the Department of Anthropology, Pennsylvania State University, USA, with a current focus on craniofacial variation using an interdisciplinary approach which combines high-resolution imaging, geometric morphometrics and wet lab techniques. Meng is an Instructor (tenure-track) in the lab of Ethylin Wang Jabs at Icahn School of Medicine at Mount Sinai, New York, USA, investigating the developmental mechanisms of birth defects, currently focusing on modelling and elucidating human malformation disorders using mice, organoids, pluripotent stem cells and bioinformatics.


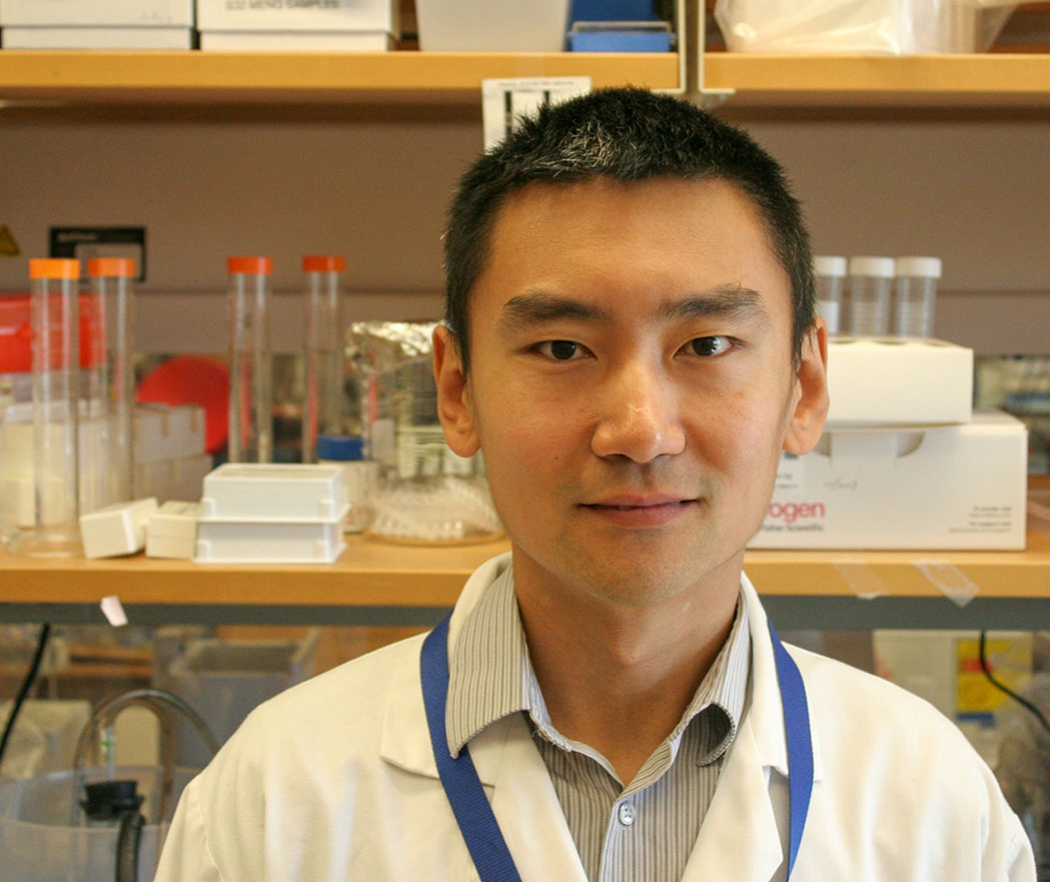


**Meng Wu**


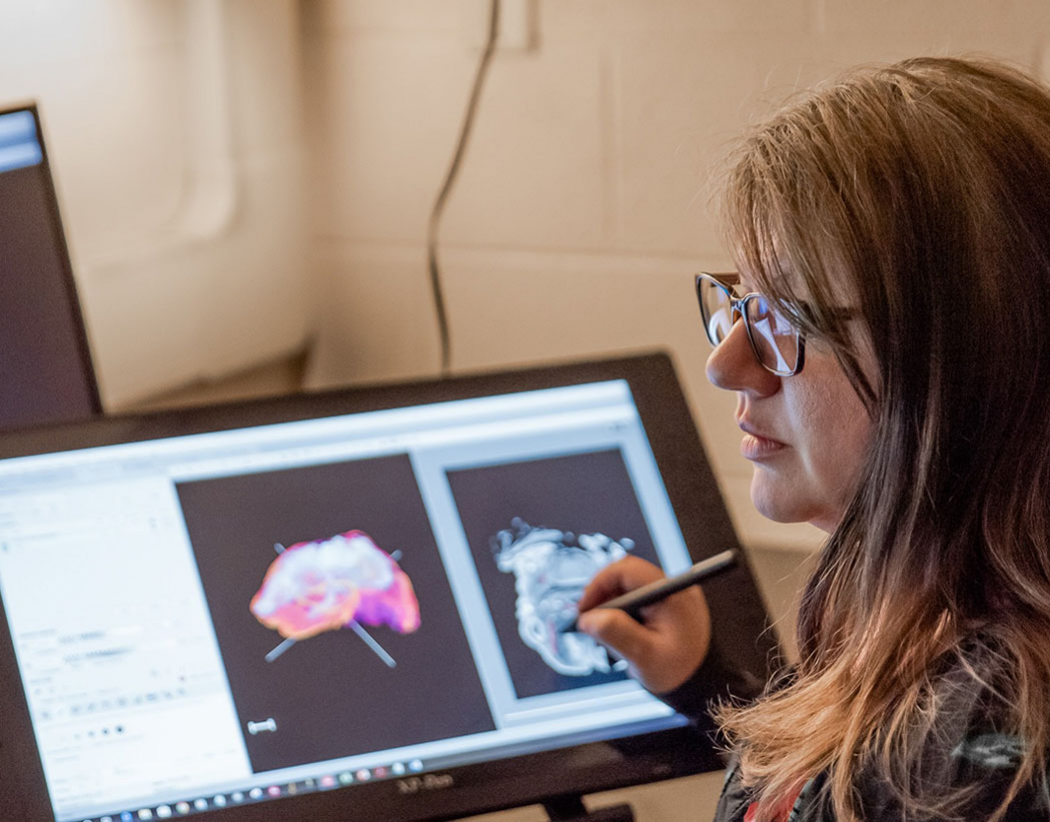


**Susan M. Motch Perrine**

**How would you explain the main findings of your paper to non-scientific family and friends?**

For this paper, we studied mice with genetic mutations that have also been found in people. Humans with certain fibroblast growth factor receptor 2 (FGFR2) mutations usually display craniosynostosis, which is the premature fusion of the skull sutures or early disappearance of the fontanels in a baby. Many organs and structures may be affected, and these individuals may have trouble eating or breathing due to abnormal growth or development of the nasal passages, mouth and throat. Surgical reconstruction of the face and other features is often the only way to improve function and may have to be repeated several times as the patient grows. Our study focused on the jaw, specifically the mandible bone and cartilage. We first scanned the heads of newborn mice to get 3D high-resolution images of the mandibles, and compared the mandibular shape, bone mineral density and bone volume in mice that carried the mutations to those of their littermates without the mutations, and found that there was a significant difference between the two groups. The posterior portion of the mandibles was most different, and we carried out several experiments to see what was happening in the jaws of mouse embryos. We used laser to capture tiny tissues in the mandible and sequenced this to find the genes expressed. We discovered increased expression of genes that are related to osteoclast (a cell that breaks down bone) activity and dysregulation of genes active in bone mineralization. We also found increased growth of certain cells that form Meckel's cartilage, which is an early component within the mandibular bone. Together, these results told us that the genetic mutations we are studying affect both cartilage formation and development of mandibular bone.

**What are the potential implications of these results for your field of research?**

Craniosynostosis syndromes present various craniofacial phenotypes. Surgical correction and reconstruction are adaptable, targeting the midfacial skeleton, dental arcade, choanae and/or airway, often requiring significant and multiple reconstructive procedures. The mandible, the major skeletal element of the lower face, is an important consideration in surgical planning and orthodontic management in craniosynostosis syndromes to address severe anomalies affecting mastication and airway anomalies. Previously, the apparent mandibular prognathism was thought to be relative to anomalies of the cranial base and severe retrusion of the midface. We provide evidence and suggest a model to show that mandibular development is directly affected by these mutations in mice. Studies such as this give us insight as to how the mandible is affected in FGFR2-related craniosynostosis syndromes and move us closer to therapeutic approaches for patients.

**What are the main advantages and drawbacks of the model system you have used as it relates to the disease you are investigating?**

Mouse models allow us to examine tissues displaying the same or a very similar phenotype to that seen in humans. Mice are genetically very similar to humans and we can easily manipulate their genomes to study conditions of great importance to medicine and health. Mouse models allow us to explore how different cells and tissues interact during embryonic as well as adult development and provide us with valuable information on tissue-specific effects of mutations causative for syndromic craniosynostosis that cannot be obtained from human studies. However, mouse models carrying a mutation we have introduced may not reproduce well or may have embryonic or early postnatal attrition or death, making it difficult to study certain stages and ages of development.

**Figure:**
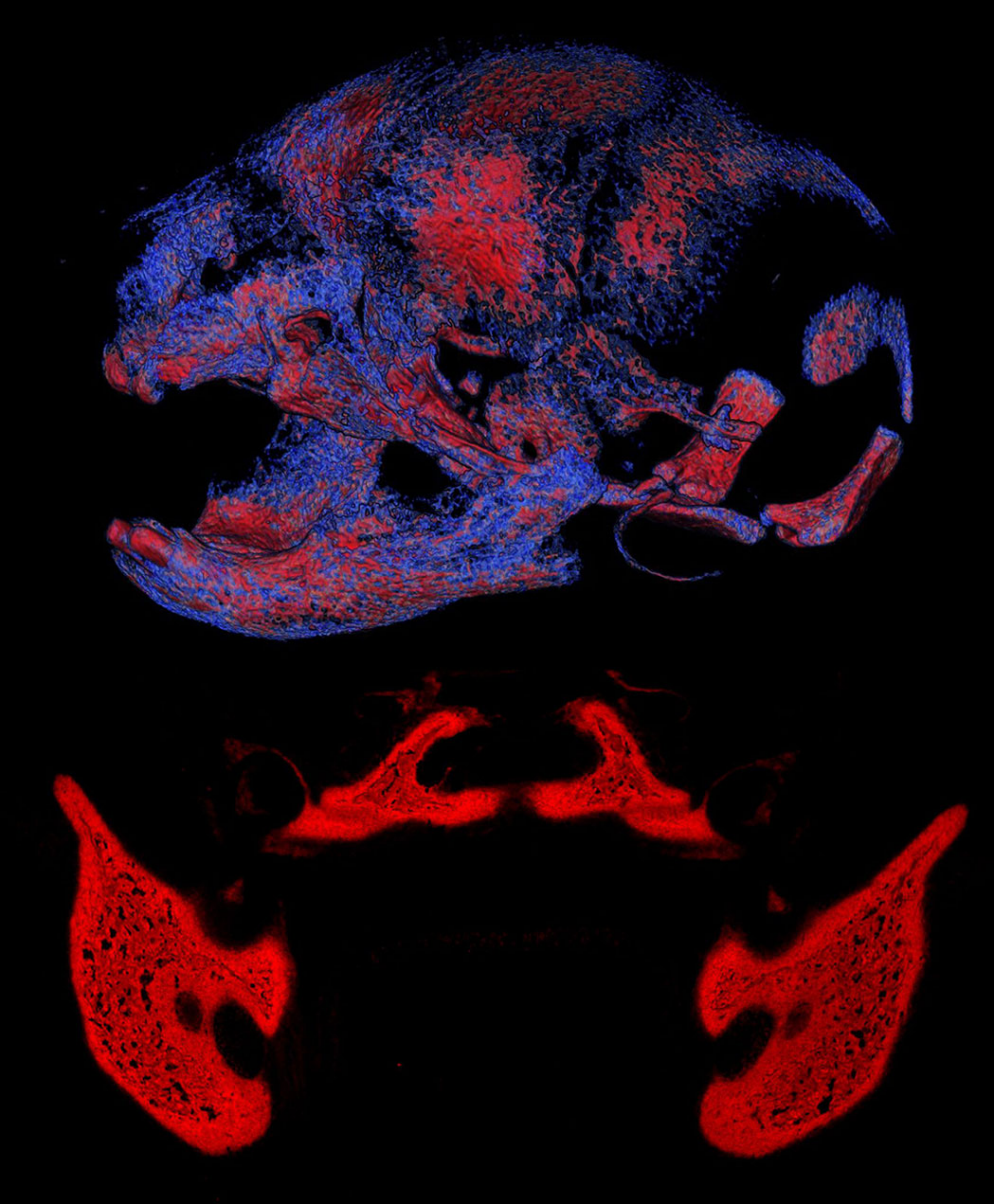
**Craniofacial tissues of an Apert syndrome mouse and wild-type embryo.** Top: volume rendering of mineralized craniofacial tissues (lateral view of skull) from µCT images of a newborn (P0) *Fgfr2^+/S252W^* Apert syndrome mouse (image by S.M.M.P.). Bottom: the osteogenic tissue in the mandibular and palatine regions of a wild-type mouse embryo at E16.5, visualized using alkaline phosphatase staining (red) (image by M.W.).

**What has surprised you the most while conducting your research?**

While variation is expected when comparing phenotypes of individuals carrying a mutation versus those without, the extreme variation we have seen in the development of certain tissues amazes us. We find it fascinating and surprising how resilient the body can be as genetic perturbations are introduced into the system. We also were surprised that the increase of osteoclastic activity was consistent to the magnitude of relative changes in mandibular morphology and histological findings in the mouse models for these different syndromes, indicating a common mechanism affecting morphogenesis in FGFR2-related mouse models.

“We find it fascinating and surprising how resilient the body can be as genetic perturbations are introduced into the system.”

**Describe what you think is the most significant challenge impacting your research at this time and how will this be addressed over the next 10 years?**

We have to say that obtaining funding and maintaining a positive public perception of animal research are among the most significant challenges impacting our research. Much of the research we are interested in is basic biology, which is necessary to understand the mechanisms through which these diseases progress as patients age so that we can attempt to develop non-surgical interventions. We believe it is very important that scientists continue to lobby for financial support of basic medical research and beyond so that we can continue to offer improvements for all types of conditions, in particular rare disorders. Education of the general public as to the usefulness of all forms of scientific research in maintaining and improving the health of society is of utmost importance now and into the future.

“Education of the general public as to the usefulness of all forms of scientific research in maintaining and improving the health of society is of utmost importance now and into the future.”

**What changes do you think could improve the professional lives of early-career scientists?**

We have excellent mentors who have nurtured our collaborations with multiple labs and taught us the grant process. We are very blessed in that regard. We feel many early-career scientists would benefit from more or multiple mentors. Very few problems are singular in nature, and we need to continue to emphasize multi-disciplinary research to further research in all our fields. Taking a multi-disciplinary approach to tackling difficult problems should be encouraged through more early-career funding opportunities that encourage collaboration between many types of researchers. More funding programmes, which are specific to early-career scientists to allow them to gain expertise in more than one discipline, would be extremely helpful. These opportunities can motivate young scientists from many different fields to work together as well as foster earlier independency in careers.

“More funding programmes, which are specific to early-career scientists to allow them to gain expertise in more than one discipline, would be extremely helpful.”

**What's next for you?**

We are continuing to investigate the mandible as well as other birth defects. We are seeking to understand more about the development of other structures of the head, within the context of craniosynostosis syndromes and beyond. We are very interested in the relationship of cartilage and bone, and in the differences in development that present upon introduction of a genetic mutation. The morphogenesis of the skull is so complex and there are still many details unknown in the skull development and pathogenesis of craniosynostosis syndromes. Are other bones and cartilages affected similarly as in the mandible in these syndromes? How exactly are the signalling pathways changed by FGFR2 mutations and can we ameliorate the phenotypes? What happens in the dysmorphogenesis of the mandible by other mutations? These are the questions that we are eager to answer next.
